# Prevalence of inflammatory bowel disease in the Australian general practice population: A cross-sectional study

**DOI:** 10.1371/journal.pone.0252458

**Published:** 2021-05-27

**Authors:** Doreen Busingye, Allan Pollack, Kendal Chidwick

**Affiliations:** Health Intelligence, NPS MedicineWise, Sydney, New South Wales, Australia; University of Oslo, NORWAY

## Abstract

The burden of inflammatory bowel disease (IBD) in Australia is increasing but national data about the current prevalence are limited. We aimed to estimate the prevalence of IBD (including Crohn’s disease, ulcerative colitis and unspecified IBD) as well as Crohn’s disease and ulcerative colitis separately in a general practice population in Australia. We also assessed risk factors associated with Crohn’s disease and ulcerative colitis. We conducted a cross-sectional study using data from MedicineInsight, a national database of general practice electronic health records, from 1 July 2017 to 30 June 2019. The prevalence of IBD was calculated and stratified by sociodemographic characteristics. Logistic regression analysis was conducted to assess risk factors associated with Crohn’s disease and ulcerative colitis. The study comprised 2,428,461 regular patients from 481 practices. The estimated crude prevalence of IBD was 653 per 100,000 patients; Crohn’s disease was 306 per 100,000 and ulcerative colitis was 334 per 100,000. Males were independently associated with a lower risk of Crohn’s disease (OR: 0.86; 95% CI: 0.81, 0.90) but a greater risk of ulcerative colitis (OR: 1.12; 95% CI: 1.06, 1.17) than females. Compared to non-smokers, patients who were current smokers were associated with a greater risk of Crohn’s disease (OR: 1.13; 95% CI: 1.04, 1.23) but a lower risk of ulcerative colitis (OR: 0.52; 95% CI: 0.47, 0.57). Other factors positively associated with both Crohn’s disease and ulcerative colitis were age (≥ 25 years), non-Indigenous status and socioeconomic advantage. Our findings provide a current estimate of the prevalence of IBD, Crohn’s disease and ulcerative colitis in a large national general practice population in Australia and an assessment of the factors associated with Crohn’s disease and ulcerative colitis. These data can assist in estimating the health burden and costs, and planning for health services.

## Introduction

Crohn’s disease (CD) and ulcerative colitis (UC), known collectively as inflammatory bowel disease (IBD), are life-long gastrointestinal disorders [1]. IBD is an important public health challenge globally and is associated with significant morbidity and reduced quality of life [2].

In recent decades, there has been a dramatic epidemiological change in the incidence of IBD in the Asia-Pacific region [3, 4]. Australia has not been spared from the increasing burden of IBD [5–7]. The few epidemiological studies from Australia show a significant incidence and prevalence of the disease [6, 7], but are limited by geographical coverage, sample size and the characteristics of the populations studied. Moreover, incidence or prevalence statistics extrapolated from limited locales and historical or international data, as done previously [5, 8], may not be reliable. To the best of our knowledge, there is no current Australian national prevalence estimate for IBD, which is important for planning health services and assessing economic costs.

The aim of this study was to estimate the prevalence of IBD (comprising CD, UC and unspecified IBD) as well as CD and UC separately in the Australian primary care setting using MedicineInsight, a large-scale national general practice database. We also assessed the sociodemographic and behavioral factors associated with CD and UC.

## Material and methods

### Study design and period

A cross-sectional study was conducted using MedicineInsight data from 1 July 2017 to 30 June 2019. Historical records outside this study period were included when identifying IBD.

### Data source

MedicineInsight is a large-scale, national general practice data program developed and managed by NPS MedicineWise with funding from the Australian Government Department of Health. The MedicineInsight programme has been described elsewhere [9]. Briefly, MedicineInsight extracts and collates longitudinal, de-identified patient health records from clinical information systems (CIS; ‘Best Practice’ or ‘MedicalDirector’) of general practices that have consented to participate in the program [10]. The data collected include patient demographics, encounters (but not progress notes), diagnoses, prescriptions and pathology tests. Progress notes, data recorded by providers about patient care in the unstructured area of the medical record, are not collected because they may contain identifiable information. The sociodemographic characteristics of regularly attending MedicineInsight patients are broadly comparable to patients who visited a GP in 2016–17 in the Medicare Benefits Schedule (MBS, a listing of services in Australia’s universal health insurance scheme) data [9].

### Study population

We included regular patients who had valid information for age and sex. We defined ‘regular’ patients as those who met the Royal Australian College of General Practitioners’ (RACGP’s) definition of an ‘active’ patient (at least three consultations with a GP at the same general practice within a two-year period). MedicineInsight data from 481 practices that met the standard data quality criteria (described elsewhere) [9] were included.

### Ascertainment of patients with IBD

A diagnosis of IBD was identified from one or more of three CIS diagnosis fields including diagnosis and medical history, the reason for encounter and the reason for prescription, using relevant free-text terms or CIS-specific codes (‘Docle’ or ‘Pyefinch’ from Best Practice or MedicalDirector).

Patients were defined as having IBD if relevant terms and synonyms of CD, UC or IBD (not specified by clinician, hereafter IBD-unspecified) were recorded in one of the three diagnosis fields, at any time in their medical records up to the end of the study (S1 Table). We further identified patients with either CD or UC only as those with relevant terms and synonyms of CD or UC recorded in one of the three diagnosis fields, at any time in their medical records up to the end of the study. For patients who had both UC and CD or IBD-unspecified recorded in their medical records, the most recent recorded diagnosis was considered as the final diagnosis [11]. Patients with a record of IBD-unspecified were only included in the overall IBD population but not the CD or UC sub-populations because it was not clear whether they had CD or UC.

### Outcome

Prevalence was defined as the proportion of the study population who had a diagnosis of IBD, CD and UC recorded up to 30 June 2019.

### Covariates

Sociodemographic characteristics comprised age (at 1 July 2019, based on year of birth), sex, Aboriginal and Torres Strait Islander status, state/territory, remoteness, Socio-Economic Indexes for Areas (SEIFA) and most recently recorded smoking status up to 30 June 2019. Information on patients’ Aboriginal or Torres Strait Islander status is recorded within the CIS and extracted into MedicineInsight using the Australian Bureau of Statistics (ABS) standard classification [12]. State/territory, remoteness and SEIFA are based on the patients’ most recently recorded residential postcodes. Remoteness was determined by the ABS geographical framework ‘Remoteness Areas’ [13], and SEIFA quintiles (1 to 5, least to most advantaged) by the ABS Index of Relative Socio-Economic Advantage and Disadvantage [14].

### Data analysis

Descriptive statistics were used to present the study outcomes, including use of percentages and associated 95% confidence intervals (CI), and means and standard deviations (SDs). Robust standard errors were used in the calculation of 95% CIs to adjust for clustering by practice. The crude prevalence of IBD was calculated and stratified by sociodemographic characteristics. Logistic regression analysis was conducted to determine risk factors associated with CD and UC. All relevant variables such as age, sex, Indigenous status, state/territory, remoteness, socioeconomic and smoking status were introduced into multivariable regression analysis in a backward stepwise elimination method and variables with a p ≤ 0.1 were retained in the final models.

A difference between groups was considered statistically significant if p < 0.05, or there was non-overlap of 95% CIs [15], where appropriate. Data management and analyses were conducted with SAS Enterprise Guide 7.1 (Cary, NC USA, 2015) and Stata 16 (College campus, TX, USA).

### Ethics

Approval to conduct this study was received on June 17, 2020 from the MedicineInsight independent external Data Governance Committee (reference number: 2020–017). The Royal Australian College of General Practitioners (RACGP) National Research and Evaluation Ethics Committee (NREEC) granted ethics approval for the standard operation and use of the MedicineInsight database by NPS MedicineWise (NREEC 17–017).

## Results

### Characteristics of the study population

There were 2,428,461 patients eligible for inclusion, representing approximately 9.7% of the Australian population [16], with a mean (SD) age of 42 (24) years and 56.1% were female (S2 Table). The demographic profiles of the study population and the national MBS data for patients in Australia who visited a GP during 2018–19 [17] are largely similar in terms of age, gender and socioeconomic status, but females are slightly overrepresented compared with the MBS patient population (52.3%) (S2 Table).

### Characteristics of patients with IBD, Crohn’s disease and ulcerative colitis

Table 1 shows the sociodemographic characteristics of patients with IBD, CD and UC. The mean age (SD) of all patients with IBD was 52 (18) years and patients with UC were on average older than those with CD (55 vs 50 years). For CD, 45.9% of patients were ex-smokers, and for UC, 51.7% (Table 1).

**Table 1:** Sociodemographic characteristics of patients with IBD, Crohn’s disease and ulcerative colitis.

Characteristic	Patients with IBD (N = 15,859)		Patients with Crohn’s disease (N = 7,442)		Patients with ulcerative colitis (N = 8,121)	
	Number	% (95% CI)	Number	% (95% CI)	Number	% (95% CI)
**Age (years)**						
Age, mean (SD)	52.4 (18.0)		49.8 (17.4)		54.9 (18.2)	
Age, median (Q1, Q3)	52 .0 (38.0, 66.0)		50.0 (36.0, 62.0)		55.0 (41.0, 69.0)	
**Age group (years)**						
0–9	27	0.17 (0.11, 0.23)	9	0.12 (0.04, 0.20)	13	0.16 (0.07, 0.25)
10–19	337	2.13 (1.88, 2.37)	208	2.79 (2.41, 3.18)	124	1.53 (1.26, 1.79)
20–29	1,453	9.16 (8.58, 9.74)	791	10.63 (9.84, 11.42)	628	7.73 (7.03, 8.43)
30–39	2,467	15.56 (14.71, 16.40)	1,302	17.50 (16.49, 18.50)	1,119	13.78 (12.68, 14.88)
40–49	2,798	17.64 (16.98, 18.30)	1,401	18.83 (17.97, 19.68)	1,352	16.65 (15.72, 17.58)
50–59	2,982	18.80 (18.12, 19.49)	1,473	19.79 (18.84, 20.75)	1,453	17.89 (16.97, 18.81)
60–69	2,716	17.13 (16.45, 17.80)	1,184	15.91 (15.04, 16.78)	1,482	18.25 (17.33, 19.17)
70–79	1,968	12.41 (11.64, 13.18)	741	9.96 (9.07, 10.85)	1,192	14.68 (13.70, 15.65)
80–89	899	5.67 (5.16, 6.17)	270	3.63 (3.14, 4.11)	611	7.52 (6.78, 8.27)
90+	212	1.34 (1.12, 1.55)	63	0.85 (0.63, 1.06)	147	1.81 (1.47, 2.15)
**Sex**						
Male	6,750	42.56 (41.67, 43.46)	2,936	39.45 (38.22, 40.69)	3,686	45.39 (44.15, 46.62)
Female	9,109	57.44 (56.54, 58.33)	4,506	60.55 (59.31, 61.78)	4,435	54.61 (53.38, 55.85)
**Indigenous status**						
Not Aboriginal and/or Torres Strait Islander	13,031	82.17 (79.32, 85.01)	6,159	82.76 (79.87, 85.65)	6,637	81.73 (78.93, 84.53)
Aboriginal and/or Torres Strait Islander	250	1.58 (1.28, 1.88)	123	1.65 (1.27, 2.03)	121	1.49 (1.16, 1.82)
Missing	2,578	16.26 (13.36, 19.15)	1,160	15.59 (12.67, 18.51)	1,363	16.78 (13.93, 19.64)
**State/territory**						
Australian Capital Territory	409	2.58 (0.90, 4.26)	188	2.53 (0.90, 4.15)	211	2.60 (0.84, 4.35)
New South Wales	5,293	33.38 (28.44, 38.31)	2,541	34.14 (29.12, 39.17)	2,659	32.74 (27.77, 37.72)
Northern Territory	95	0.60 (0.15, 1.05)	40	0.54 (0.12, 0.95)	50	0.62 (0.13, 1.10)
Queensland	2,791	17.60 (13.70, 21.50)	1,182	15.88 (12.26, 19.51)	1,561	19.22 (14.97, 23.48)
South Australia	610	3.85 (1.76, 5.93)	320	4.30 (2.00, 6.60)	285	3.51 (1.54, 5.48)
Tasmania	1,366	8.61 (5.14, 12.09)	638	8.57 (5.11, 12.04)	703	8.66 (5.11, 12.21)
Victoria	3,689	23.26 (17.70, 28.83)	1,735	23.31 (17.58, 29.05)	1,883	23.19 (17.69, 28.68)
Western Australia	1,606	10.13 (6.88, 13.38)	798	10.72 (7.34, 14.10)	769	9.47 (6.27, 12.67)
**Remoteness**						
Major city	9,458	59.64 (54.23, 65.05)	4,462	59.96 (54.45, 65.46)	4,823	59.39 (53.92, 64.85)
Inner regional	4,427	27.91 (23.24, 32.59)	2,073	27.86 (23.04, 32.67)	2,284	28.12 (23.39, 32.86)
Outer regional	1,767	11.14 (8.49, 13.80)	796	10.70 (8.12, 13.27)	925	11.39 (8.59, 14.19)
Remote/very remote	207	1.31 (0.66, 1.95)	111	1.49 (0.69, 2.29)	89	1.10 (0.55, 1.64)
**Socioeconomic status (SEIFA quintiles)**						
1 (least advantaged)	2,315	14.60 (11.92, 17.27)	1,075	14.45 (11.74, 17.15)	1,193	14.69 (11.88, 17.50)
2	2,631	16.59 (13.92, 19.26)	1,271	17.08 (14.35, 19.81)	1,310	16.13 (13.42, 18.84)
3	4,004	25.25 (21.97, 28.52)	1,919	25.79 (22.38, 29.19)	2,020	24.87 (21.52, 28.22)
4	3,304	20.83 (18.28, 23.39)	1,517	20.38 (17.77, 22.99)	1,728	21.28 (18.57, 23.99)
5 (most advantaged)	3,586	22.61 (18.88, 26.34)	1,653	22.21 (18.37, 26.05)	1,858	22.88 (19.11, 26.65)
Missing	19	0.12 (0.00, 0.25)	7	0.09 (0.00, 0.20)	12	0.15 (0.00, 0.32)
**Smoking status**						
Non-smoker	5,212	32.86 (29.50, 36.23)	2,371	31.86 (28.47, 35.25)	2,730	33.62 (30.12, 37.11)
Ex-smoker	7,752	48.88 (45.79, 51.97)	3,418	45.93 (42.81, 49.05)	4,202	51.74 (48.48, 55.00)
Current smoker	1,652	10.42 (9.55, 11.28)	1,061	14.26 (13.14, 15.37)	561	6.91 (6.05, 7.77)
Missing	1,243	7.84 (7.05, 8.63)	592	7.95 (7.08, 8.83)	628	7.73 (6.83, 8.64)

CI: confidence interval; IBD: Inflammatory bowel disease; SD: standard deviation; SEIFA: Socio-Economic Indexes for Areas; Q1: 25^th^ percentile; Q3: 75^th^ percentile.

### Prevalence of IBD, Crohn’s disease and ulcerative colitis

Among the study population, 15,859 patients (653 per 100,000) were identified as having IBD (including CD, UC and IBD-unspecified). The overall crude prevalence per 100,000 of CD, UC and IBD-unspecified was 306 (n = 7,442), 334 (n = 8,121) and 12 (n = 296), respectively (Table 2). Prevalence of IBD, CD and UC was significantly greater in patients aged ≥ 20 than those aged < 20 years and in non-Indigenous than Aboriginal and/or Torres Strait Islander patients. Prevalence of CD was significantly greater for females (331) than males (275) but prevalence of UC was similar for both sexes (Table 2). Compared with the overall IBD prevalence for the study population (653), the IBD prevalence was significantly greater for patients resident in South Australia (908) and Tasmania (831), and lower in the Northern Territory (330). Similar findings were observed for CD and UC.

**Table 2:** Prevalence (per 100,000) of IBD, Crohn’s disease and ulcerative colitis, stratified by sociodemographic characteristics and smoking status.

Characteristic	Patients with IBD		Patients with CD		Patients with UC	
	per 100,000	95% CI	per 100,000	95% CI	per 100,000	95% CI
**Overall**	653	623, 684	306	292, 321	334	317, 352
**Sex**						
Male	633	598, 668	275	258, 293	346	325, 366
Female	669	638, 699	331	315, 346	326	308, 343
**Age group (years)**						
0–9	9	6, 13	3	1, 5	5	2, 7
10–19	151	131, 170	93	79, 107	55	45, 66
20–29	503	450, 556	274	242, 305	217	191, 244
30–39	757	707, 807	399	366, 433	343	318, 368
40–49	902	848, 956	452	420, 483	436	404, 468
50–59	954	907, 1002	471	443, 500	465	434, 496
60–69	914	875, 953	398	376, 421	499	469, 528
70–79	850	806, 894	320	294, 346	515	484, 546
80–89	776	720, 832	233	203, 263	527	483, 572
90+	603	518, 687	179	136, 223	418	346, 490
**Indigenous status**						
Not Aboriginal and/or Torres Strait Islander	678	645, 711	321	304, 337	345	326, 364
Aboriginal and/or Torres Strait Islander	337	269, 404	166	128, 203	163	125, 201
**State/territory**						
Australian Capital Territory	673	563, 783	309	251, 368	347	282, 412
New South Wales	643	606, 679	308	289, 328	323	302, 344
Northern Territory	330	250, 411	139	86, 193	174	133, 214
Queensland	596	556, 637	253	233, 272	334	305, 362
South Australia	908	805, 1010	476	410, 542	424	370, 478
Tasmania	831	768, 893	388	349, 427	428	390, 466
Victoria	689	587, 791	324	276, 372	352	296, 407
Western Australia	574	523, 626	285	261, 310	275	239, 311
**Remoteness**						
Major city	648	615, 681	306	288, 323	330	312, 349
Inner regional	687	629, 746	322	297, 347	355	318, 392
Outer regional	602	540, 665	271	240, 302	315	278, 352
Remote/very remote	666	533, 798	357	270, 444	286	212, 361
**Socioeconomic status (SEIFA quintiles)**						
1 (least advantaged)	637	592, 682	296	274, 317	328	295, 361
2	630	574, 685	304	275, 334	314	283, 344
3	641	604, 678	307	287, 328	324	301, 346
4	655	613, 698	301	279, 323	343	316, 369
5 (most advantaged)	693	645, 742	320	292, 348	359	333, 385
**Smoking status**						
Non-smoker	680	648, 711	309	292, 326	356	337, 375
Ex-smoker	808	747, 868	356	329, 383	438	403, 473
Current smoker	619	576, 662	398	365, 431	210	191, 230

CI: confidence interval; CD: Crohn’s disease; IBD: Inflammatory bowel disease; SD: standard deviation; SEIFA: Socio-Economic Indexes for Areas; Q1: 25^th^ percentile; Q3: 75^th^ percentile; UC: ulcerative colitis.

**Fig 1:**
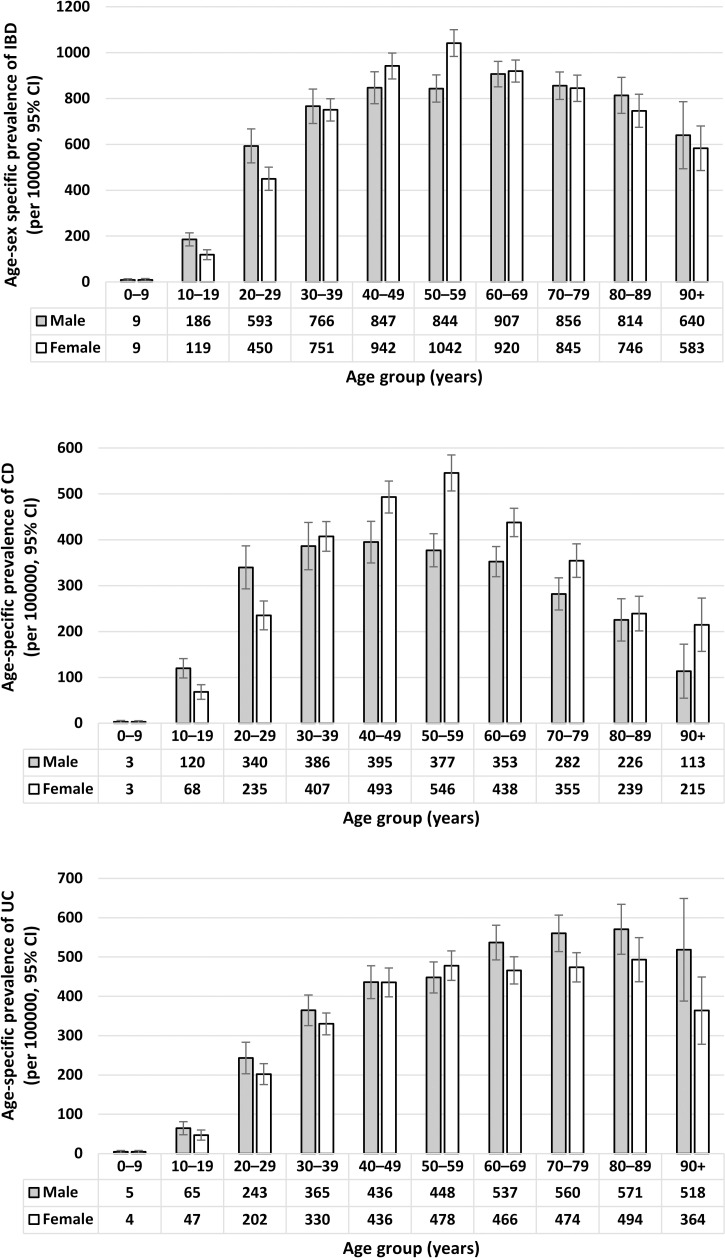
Age-sex specific prevalence (per 100,000) of IBD, Crohn’s disease and ulcerative colitis. Non-overlap of 95% CIs indicates significant differences between males and females for each age group. IBD: Inflammatory bowel disease; CD: Crohn’s disease; UC: Ulcerative colitis.

There were significant differences in the prevalence of IBD between males and females among patients aged 10–29 and 50–59 years (Fig 1 and S3 Table). For patients aged 10–29 years, the prevalence of CD was greater in males than females but was greater in females than males for patients aged 40–79 years (Fig 1 and S3 Table). In contrast to CD, there was no significant difference between males and females in the prevalence of UC for most age groups, except for the 70–79 years where the prevalence was greater among males than females (Fig 1 and S3 Table).

### Factors associated with Crohn’s disease and ulcerative colitis

Table 3 shows factors associated with CD and UC after adjustment using multivariable logistic regression. Patients who were aged ≥ 25 years, ex-smokers, and the socioeconomically advantaged had a greater risk of having CD or UC compared to those aged ≤ 24 years, non-smokers and the socioeconomically disadvantaged, respectively. Aboriginal and/or Torres Strait Islander patients had a lower risk of both CD (OR: 0.62; 95% CI: 0.52, 0.76) and UC (OR: 0.74; 95% CI: 0.62, 0.90). Compared to females, being male was associated with a lower risk of CD (OR: 0.86; 95% CI: 0.81, 0.90) but a greater risk of UC (OR: 1.12; 95% CI: 1.06, 1.17). Interestingly, compared to non-smokers, patients who were current smokers were associated with a greater risk of CD (OR: 1.13; 95% CI: 1.04, 1.23) but a lower risk of UC (OR: 0.52; 95% CI: 0.47, 0.57). Ex-smokers had a greater risk of both CD and UC than non-smokers. Variations in the risk of CD and UC were observed by state/territory.

**Table 3:** Factors associated with Crohn’s disease and ulcerative colitis.

Factors	Crohn’s disease				Ulcerative colitis			
	Univariable analysis		Multivariable analysis^a^		Univariable analysis		Multivariable analysis^a^	
	OR (95% CI)	p-value	OR (95% CI)	p-value	OR (95% CI)	p-value	OR (95% CI)	p-value
Male	0.83 (0.79, 0.87)	<0.001	0.86 (0.81, 0.90)	<0.001	1.06 (1.02, 1.11)	0.01	1.12 (1.06, 1.17)	<0.001
Age group (years)								
0–24	1.00		1.00		1.00		1.00	
25–44	4.82 (4.39, 5.30)	<0.001	3.94 (3.50, 4.43)	<0.001	6.55 (5.85, 7.34)	<0.001	5.75 (5.00, 6.62)	<0.001
45–64	5.83 (5.31, 6.40)	<0.001	4.67 (4.16, 5.25)	<0.001	8.72 (7.80, 9.74)	<0.001	7.40 (6.44, 8.50)	<0.001
≥65	3.81 (3.45, 4.20)	<0.001	3.02 (2.68, 3.41)	<0.001	9.50 (8.49, 10.62)	<0.001	7.61 (6.62, 8.75)	<0.001
Aboriginal and/or Torres Strait Islander	0.52 (0.43, 0.62)	<0.001	0.62 (0.52, 0.76)	<0.001	0.47 (0.39, 0.56)	<0.001	0.74 (0.62, 0.90)	0.002
Smoking status								
Non-smoker	1.00		1.00		1.00		1.00	
Current smoker	1.29 (1.20, 1.38)	<0.001	1.13 (1.04, 1.23)	0.003	0.59 (0.54, 0.65)	<0.001	0.52 (0.47, 0.57)	<0.001
Ex-smoker	1.15 (1.09, 1.21)	<0.001	1.13 (1.06, 1.19)	<0.001	1.23 (1.17, 1.29)	<0.001	1.13 (1.07, 1.20)	<0.001
Remoteness								
Major city	1.00		–	–	1.00		–	–
Inner regional	1.05 (1.00, 1.11)	0.05	–	–	1.07 (1.02, 1.13)	0.01	–	–
Outer regional	0.89 (0.82, 0.96)	0.002	–	–	0.95 (0.89, 1.02)	0.19	–	–
Remote/very remote	1.17 (0.97, 1.41)	0.11	–	–	0.87 (0.70, 1.07)	0.18	–	–
Socioeconomic status (SEIFA quintiles)								
1 (least advantaged)	1.00		1.00		1.00		1.00	
2	1.03 (0.95, 1.12)	0.50	1.07 (0.97, 1.17)	0.17	0.95 (0.88, 1.03)	0.25	1.04 (0.95, 1.14)	0.37
3	1.04 (0.96, 1.12)	0.32	1.09 (1.00, 1.19)	0.05	0.99 (0.92, 1.06)	0.69	1.06 (0.98, 1.15)	0.16
4	1.02 (0.94, 1.10)	0.43	1.14 (1.03, 1.25)	0.01	1.04 (0.97, 1.12)	0.25	1.18 (1.08, 1.28)	<0.001
5 (most advantaged)	1.08 (1.00, 1.17)	0.05	1.19 (1.07, 1.31)	0.001	1.09 (1.02, 1.18)	0.02	1.21 (1.10, 1.32)	<0.001
State/territory								
New South Wales	1.00		1.00		1.00		1.00	
Australian Capital Territory	1.00 (0.86, 1.16)	0.97	0.91 (0.77, 1.08)	0.28	1.08 (0.94, 1.24)	0.31	0.93 (0.79, 1.10)	0.40
Northern Territory	0.45 (0.33, 0.62)	<0.001	0.40 (0.28, 0.58)	<0.001	0.54 (0.41, 0.71)	<0.001	0.54 (0.40, 0.73)	<0.001
Queensland	0.82 (0.76, 0.88)	<0.001	0.80 (0.74, 0.87)	<0.001	1.03 (0.97, 1.10)	0.31	1.02 (0.95, 1.10)	0.60
South Australia	1.55 (1.38, 1.74)	<0.001	1.53 (1.34, 1.74)	<0.001	1.31 (1.16, 1.49)	<0.001	1.22 (1.06, 1.40)	0.01
Tasmania	1.26 (1.15, 1.37)	<0.001	1.30 (1.17, 1.44)	<0.001	1.33 (1.22, 1.44)	<0.001	1.40 (1.27, 1.54)	<0.001
Victoria	1.05 (0.99, 1.12)	0.12	0.96 (0.89, 1.03)	0.25	1.09 (1.03, 1.16)	0.004	1.05 (0.98, 1.12)	0.20
Western Australia	0.92 (0.85, 1.00)	0.05	0.93 (0.84, 1.02)	0.10	0.85 (0.79, 0.92)	<0.001	0.88 (0.81, 0.97)	0.01

CI: confidence interval; OR: odds ratio; SEIFA: Socio-Economic Indexes for Areas

^a^ Backward stepwise elimination method adjusted for age, sex, Aboriginal and/or Torres Strait Islander status, smoking status, remoteness, socioeconomic status and state/territory.

## Discussion

We have demonstrated the prevalence of IBD overall (including CD, UC and IBD- unspecified), and of CD and UC separately, in a large national Australian general practice population. The unadjusted prevalence of IBD, CD and UC at the end of the 2-year study period was estimated to be 653 per 100,000, 306 and 334, respectively. Our findings suggest higher rates than previously reported in regional population-based studies from Australia [6, 7]. Investigators of a 2010–11 study from Victoria showed the crude prevalence of IBD to be 344.6 per 100,000 (197.3 for CD and 136.0 per 100,000 for UC) [7]. In a 2013–14 population-based study from Tasmania, the crude prevalence per 100,000 of IBD, CD and UC was 335.0, 170.3, and 156.5, respectively [6]. Estimates observed in our study are similar to those reported in some European countries [11, 18]. A recent study conducted in primary care patients in the UK showed that the prevalence of IBD, CD and UC in 2018 was 725, 276 and 397 per 100,000, respectively [11]. A German study, utilizing administrative data, found that the prevalence of IBD in 2010 was 744 per 100,000 with 322 for CD and 412 for UC [18].

The higher prevalence in our study may reflect the increasing incidence of IBD [6, 7]; improved life expectancies of patients with IBD; variations in the source population (e.g., general practice patient population versus general population); and differences in data collection. As IBD is a chronic relapsing condition, and some clinicians might not routinely record a diagnosis of an existing chronic condition at every clinical encounter, we assessed lifetime prevalence where patients who ever had a record of IBD, CD and UC in their clinical record were included. Lifetime prevalence estimates might be higher than cross-sectional point (current) prevalence estimates if patients who are in long term remission are included.

While some Australian reports have shown higher rates of CD than UC [6, 7], our study shows slightly higher rates of UC than CD. This finding aligns with data presented in a 2006 report commissioned by the Australian Crohn’s and Colitis Association [8], and is consistent with some reports from Europe [18, 19] and USA [20].

Our findings also indicate variations in prevalence regarding sex, age group, Indigenous status, smoking status, socioeconomic status and state/territory. Consistent with other Australian studies [6], we found that the prevalence of CD is greater among females than males but the prevalence of UC is similar for both sexes. While data about sex-based differences in IBD are conflicting [21], our findings show that being male is independently associated with a lower risk of CD but a greater risk of UC.

The finding that Aboriginal and/or Torres Strait Islanders have a lower risk for developing IBD is consistent with other reports that have demonstrated lower risk of IBD in Indigenous populations [22–24]. Data from the Australian Paediatric and Adolescent IBD Database indicate lower rates of IBD in children of Aboriginal and Torres Strait Islander descent compared to non-Indigenous children [24]. Evidence from New Zealand demonstrates a lower risk of IBD among the Maori and Pacific Island people compared to Caucasians [22, 23]. The differences in prevalence and risk of IBD between ethnic groups may be attributable to genetic susceptibilities and environmental factors [25, 26].

This analysis further confirms the well-established paradoxical association between current smoking with CD and UC. As has been documented previously [23, 27, 28], our results indicate that current smoking is independently associated with a greater risk of CD but a lower risk of UC. Investigators of another Australian study found that smoking was positively associated with CD and negatively associated with UC among Caucasian Australians [28]. The reasons for the opposing effects of smoking on CD and UC are not clear, but several mechanisms through which smoking is said to influence the aetiology of IBD have been suggested, including epigenetic alterations, disruption in intestinal microbiota, integrity of the intestinal epithelium and the immune system [27].

The finding that socioeconomic advantage is associated with an increased risk of IBD is consistent with observations from other investigators [23]. This finding is not surprising as IBD has traditionally been regarded as a disease of high-income countries because of a higher prevalence observed in these countries compared to developing countries. Data from the global burden of disease study show that locations with high sociodemographic index (SDI) have the highest age-standardized prevalence of IBD, while those with low SDI have the lowest prevalence [4].

Regional variations in the risk of CD and UC may reflect differences in population characteristics (e.g., genetic and demographic factors, and environmental exposures) and area characteristics (e.g., remoteness and socioeconomic factors), across states/territories. Nevertheless, the high prevalence of IBD in Tasmania and South Australia compared with some other states/territories may require further investigation.

The strengths of this analysis include the size and national coverage of the MedicineInsight data. To the best of our knowledge, this is the first large national epidemiological study to present prevalence estimates for IBD in Australia. Because MedicineInsight is an open cohort and patients in Australia can visit multiple general practices, we used a cohort of regularly attending patients, likely to be receiving most of their care at a MedicineInsight practice, to help improve data quality. The use of MedicineInsight records containing GP-identified diagnoses is likely to be more accurate than self-reported health surveys.

This study is subject to a number of limitations, in addition to those inherent in routinely collected data, described elsewhere [9]. First, for privacy reasons, MedicineInsight does not include data from progress notes, which may contain further clinical information. Second, because data used are from general practice patients, this limits generalizability of the results to the general population. However, this MedicineInsight cohort of regularly attending patients is largely representative of the national MBS patient population, although minor demographic differences may have impacted our estimates, with female patients slightly overrepresented compared with MBS data. As the prevalence of CD is greater among females this could lead to a slight overestimate of the true CD (and IBD) patient prevalence. Third, as IBD is a chronic relapsing condition, we defined patients as those who have ever had a record of IBD in their clinical record. This may overestimate the current prevalence of IBD where patients are in long term remission. Fourth, because this was an observational study, this precludes us from drawing firm conclusions about temporal and causal relationships. Finally, although the prevalence estimates of several conditions derived from MedicineInsight are comparable with other studies and national estimates [17, 29], MedicineInsight data may be further refined by ongoing validation studies.

## Conclusions

Our findings provide a current estimate of the prevalence of IBD, CD and UC in a large national general practice population in Australia and an assessment of the factors associated with CD and UC. These data can assist in estimating the health burden and costs, and planning for health services as well as strategies to reduce the burden of IBD.

## Supporting information

S1 Table Relevant search terms and synonyms for Crohn’s disease, ulcerative colitis and unspecified IBD. (DOCX)

S2 Table Sociodemographic characteristics of patients in the main study cohort (2017–2019) compared to MBS national data (2018–2019). (DOCX)

S3 Table Age-sex specific prevalence (per 100,000) of IBD, Crohn’s disease and ulcerative colitis. (DOCX)
