# Supplementary material for: Prevalence of inflammatory bowel disease in the Australian general practice population: A cross-sectional study
Source: PLoS One. 2021 May 27;16(5):e0252458. doi: 10.1371/journal.pone.0252458 (PMC8158877; doi:10.1371/journal.pone.0252458)
Supplement: S1 Table — (DOCX) [file pone.0252458.s001.docx]

**S1 Table. Relevant search terms and synonyms for Crohn’s disease, ulcerative colitis and unspecified IBD**

| **Condition** | **Search terms examples** |
| --- | --- |
| Crohn’s disease |  |
|  | Crohn’s disease |
|  | Crohn disease |
|  | Crohns |
|  | Chrons |
|  | Granulomatous colitis |
|  | Regional enteritis |
| Ulcerative colitis |  |
|  | Ulcerative colitis |
|  | Colitis - ulcerative |
|  | Colitis, ulcerative |
|  | Ulcerative c |
| IBD unspecified |  |
|  | Inflammatory bowel disease |
|  | IBD |
|  | Inflamatory bowel |
|  | Inflammatory bowel |
