# Supplementary material for: Prevalence of inflammatory bowel disease in the Australian general practice population: A cross-sectional study
Source: PLoS One. 2021 May 27;16(5):e0252458. doi: 10.1371/journal.pone.0252458 (PMC8158877; doi:10.1371/journal.pone.0252458)
Supplement: S2 Table — (DOCX) [file pone.0252458.s002.docx]

**S2 Table.** **Sociodemographic characteristics of patients in the main study cohort (2017–2019) compared to MBS national data (2018–2019)**

| Characteristic | | Patients in the study cohort  (N = 2,428,461) | | Australian national data (MBS)^a^ 2018–19  (N = 21,942,493) |
| --- | --- | --- | --- | --- |
|  |  | Number | % | % |
| **Age (years)** | |  |  |  |
|  | Age, mean (SD) | 42.4 (24.2) |  | - |
|  | Age, median (Q1, Q3) | 42.0 (23.0, 62.0) |  | - |
| **Age group (years)** | |  |  |  |
|  | 0–9 | 287,024 | 11.82 (11.35, 12.29) | 12.9 |
|  | 10–19 | 223,971 | 9.22 (8.97, 9.48) | 10.9 |
|  | 20–29 | 288,996 | 11.90 (11.04, 12.76) | 12.2 |
|  | 30–39 | 326,070 | 13.43 (12.78, 14.07) | 14.0 |
|  | 40–49 | 310,225 | 12.77 (12.53, 13.02) | 13.2 |
|  | 50–59 | 312,481 | 12.87 (12.63, 13.11) | 12.8 |
|  | 60–69 | 297,225 | 12.24 (11.79, 12.69) | 11.3 |
|  | 70–79 | 231,463 | 9.53 (8.90, 10.16) | 8.0 |
|  | 80–89 | 115,844 | 4.77 (4.38, 5.16) | 3.8 |
|  | 90+ | 35,162 | 1.45 (1.31, 1.59) | 0.9 |
| **Sex** | |  |  |  |
|  | Male | 1,066,387 | 43.91 (43.47, 44.35) | 47.7 |
|  | Female | 1,362,074 | 56.09 (55.65, 56.53) | 52.3 |
| **Indigenous status^b^** | |  |  |  |
|  | Not Aboriginal and/or Torres Strait Islander | 1,921,422 | 79.12 (76.54, 81.71) | 91.2 |
|  | Aboriginal and/or Torres Strait Islander | 74,272 | 3.06 (2.45, 3.66) | 2.8 |
|  | Missing | 432,767 | 17.82 (15.19, 20.45) | 6.0 |
| **State/territory** | |  |  |  |
|  | Australian Capital Territory | 60,768 | 2.50 (0.92, 4.08) | 1.7 |
|  | New South Wales | 823,811 | 33.92 (28.85, 39.00) | 32.1 |
|  | Northern Territory | 28,764 | 1.18 (0.25, 2.12) | 0.9 |
|  | Queensland | 468,104 | 19.28 (14.96, 23.59) | 20.2 |
|  | South Australia | 67,220 | 2.77 (1.27, 4.26) | 7.0 |
|  | Tasmania | 164,455 | 6.77 (3.91, 9.63) | 2.2 |
|  | Victoria | 535,614 | 22.06 (15.68, 28.43) | 25.6 |
|  | Western Australia | 279,725 | 11.52 (7.86, 15.18) | 10.4 |
| **Remoteness** | |  |  |  |
|  | Major city | 1,459,892 | 60.12 (54.35, 65.88) | 71.5 |
|  | Inner regional | 643,978 | 26.52 (21.66, 31.38) | 12.3 |
|  | Outer regional | 293,495 | 12.09 (9.18, 14.99) | 12.3 |
|  | Remote/very remote | 31,096 | 1.28 (0.64, 1.92) | 3.9 |
| **Socioeconomic status (SEIFA quintiles)** | |  |  |  |
|  | 1 (most disadvantaged) | 363,405 | 14.96 (12.32, 17.61) | 15.8 |
|  | 2 | 417,858 | 17.21 (14.31, 20.10) | 16.1 |
|  | 3 | 624,463 | 25.71 (22.48, 28.94) | 19.7 |
|  | 4 | 504,188 | 20.76 (18.29, 23.23) | 20.8 |
|  | 5 (most advantaged) | 517,225 | 21.30 (17.81, 24.79) | 27.6 |
|  | Missing | 1,322 | 0.05 (0.00, 0.11) | - |
| **Smoking status** | |  |  |  |
|  | Never | 766,985 | 31.58 (27.83, 35.34) | - |
|  | Ex-smoker | 959,736 | 39.52 (35.96, 43.08) | - |
|  | Current smoker | 266,782 | 10.99 (10.12, 11.85) | - |
|  | Missing | 434,958 | 17.91 (16.66, 19.16) | - |

CI: confidence interval; MBS: Medicare Benefits Schedule; SD: standard deviation; SEIFA: Socio-economic Indexes for Areas; Q1: 25th percentile; Q3: 75th percentile.

^a^ MBS data for Australians who visited a general practitioner at least once between 1 July 2018 and 30 June 2019. <https://www.nps.org.au/assets/Report-2018-19-GPIR-final-signed-off-by-DOH.pdf>

^b^ National estimate is from the Census of Population and Housing - Counts of Aboriginal and Torres Strait Islander Australians, 2017. <http://www.abs.gov.au/AUSSTATS/abs@.nsf/Lookup/2075.0Main+Features12016?OpenDocument>
