# Supplementary material for: Prevalence of inflammatory bowel disease in the Australian general practice population: A cross-sectional study
Source: PLoS One. 2021 May 27;16(5):e0252458. doi: 10.1371/journal.pone.0252458 (PMC8158877; doi:10.1371/journal.pone.0252458)
Supplement: S3 Table — (DOCX) [file pone.0252458.s003.docx]

**S3 Table.** **Age-sex specific prevalence (per 100,000) of IBD, Crohn’s disease and ulcerative colitis**

| Age group (years) | | Males | | Females | |
| --- | --- | --- | --- | --- | --- |
|  |  | Number | per 100,000  (95% CI) | Number | per 100,000  (95% CI) |
| **IBD^a^** | | | | | |
|  | 0–9 | 14 | 9 (5, 14) | 13 | 9 (4, 15) |
|  | 10–19 | 198 | 186 (157, 214) | 139 | 119 (97, 140) |
|  | 20–29 | 632 | 593 (519, 668) | 821 | 450 (399, 500) |
|  | 30–39 | 952 | 766 (691, 841) | 1,515 | 751 (702, 799) |
|  | 40–49 | 1,111 | 847 (777, 917) | 1,687 | 942 (886, 999) |
|  | 50–59 | 1,163 | 844 (784, 903) | 1,819 | 1042 (983, 1100) |
|  | 60–69 | 1,245 | 907 (851, 962) | 1,471 | 920 (872, 968) |
|  | 70–79 | 941 | 856 (797, 916) | 1,027 | 845 (788, 902) |
|  | 80–89 | 415 | 814 (736, 892) | 484 | 746 (674, 819) |
|  | 90+ | 79 | 640 (493, 787) | 133 | 583 (486, 680) |
| **Crohn’s disease** | | | | | |
|  | 0–9 | 5 | 3 (0, 6) | <5^b^ | 3 (0, 6) |
|  | 10–19 | 128 | 120 (99, 141) | 80 | 68 (52, 84) |
|  | 20–29 | 362 | 340 (293, 387) | 429 | 235 (204, 267) |
|  | 30–39 | 480 | 386 (335, 438) | 822 | 407 (375, 440) |
|  | 40–49 | 518 | 395 (350, 440) | 883 | 493 (458, 528) |
|  | 50–59 | 520 | 377 (341, 413) | 953 | 546 (506, 585) |
|  | 60–69 | 484 | 353 (320, 385) | 700 | 438 (407, 469) |
|  | 70–79 | 310 | 282 (247, 317) | 431 | 355 (318, 391) |
|  | 80–89 | 115 | 226 (179, 272) | 155 | 239 (201, 277) |
|  | 90+ | 14 | 113 (55, 172) | 49 | 215 (157, 273) |
| **Ulcerative colitis** | | | | | |
|  | 0–9 | 7 | 5 (1, 8) | 6 | 4 (1, 8) |
|  | 10–19 | 69 | 65 (48, 81) | 55 | 47 (34, 60) |
|  | 20–29 | 259 | 243 (203, 283) | 369 | 202 (176, 229) |
|  | 30–39 | 453 | 365 (326, 404) | 666 | 330 (302, 358) |
|  | 40–49 | 572 | 436 (395, 478) | 780 | 436 (399, 472) |
|  | 50–59 | 618 | 448 (409, 488) | 835 | 478 (441, 516) |
|  | 60–69 | 737 | 537 (493, 581) | 745 | 466 (431, 501) |
|  | 70–79 | 616 | 560 (514, 607) | 576 | 474 (437, 511) |
|  | 80–89 | 291 | 571 (507, 634) | 320 | 494 (437, 550) |
|  | 90+ | 64 | 518 (388, 649) | 83 | 364 (278, 449) |

CI: confidence interval; IBD: Inflammatory bowel disease.

^a^ IBD includes patients with Crohn’s disease, ulcerative colitis or IBD-unspecified.

^b^ Cell sizes less than five are suppressed to maintain patient confidentiality.
